# Cortical substrates of cue-reactivity in multiple substance dependent populations: transdiagnostic relevance of the medial prefrontal cortex

**DOI:** 10.1038/s41398-018-0220-9

**Published:** 2018-09-07

**Authors:** Colleen A. Hanlon, Logan T. Dowdle, Nicole B. Gibson, Xingbao Li, Sarah Hamilton, Melanie Canterberry, Michaela Hoffman

**Affiliations:** 1 Department of Psychiatry and Behavioral Sciences Medical, University of South Carolina, Charleston, SC 29425 USA; 2 Ralph H. Johnson VA Medical Center, Charleston, SC 29425 USA

## Abstract

Elevated drug-cue elicited brain activity is one of the most widely cited, transdiagnostically relevant traits of substance dependent populations. These populations, however, are typically studied in isolation. The goal of this study was to prospectively investigate the spatial topography of drug-cue reactivity in a large set of individuals dependent on either cocaine, alcohol, or nicotine. Functional MRI data was acquired from 156 substance dependent individuals (55 cocaine, 53 alcohol, and 48 nicotine) as they performed a standardized drug-cue exposure task. Clusters of significant activation to drug-cues relative to neutral cues (‘hot spots’) were isolated for each individual. K-means clustering was used to classify the spatial topography of the hotspots in the data set. The percentage of hotspots that would be reached at several distances (2–5 cm) of transcranial magnetic stimulation (TMS) were calculated. One hundred and three participants had at least one cluster of significant frontal cortex activity (66%). K-means revealed 3 distinct clusters within the medial prefrontal cortex (MPFC), left inferior frontal gyrus/insula, right premotor cortex. For the group as a whole (and for alcohol users and nicotine users independently), medial prefrontal cortex (BA 10) was the location of the greatest number of hotspots. The frontal pole was cortical location closest to the largest percentage of hotspots. While there is individual variability in the location of the cue-elicited ‘hot spot’ these data demonstrate that elevated BOLD signal to drug cues in the MPFC may be a transdiagnostic endophenotype of addiction which may also be a fruitful neuromodulation target.

## Introduction

Elevated drug-cue elicited brain activity is one of the most widely cited, transdiagnostically relevant traits of current substance dependent populations. Many studies investigating cue-reactivity in either cocaine, nicotine, or alcohol dependent populations have independently demonstrated that drug-cues evoke elevated activity in the medial prefrontal cortex, anterior cingulate, and insula cortex. Most of these studies focus on one substance using class, however, and therefore it is difficult to distinguish which aspects of cue-reactivity are transdiagnostic biomarkers of the addiction process versus those which are specific to alcohol, cocaine, or nicotine dependence.

Several retrospective meta-analyses have demonstrated that the medial prefrontal cortex and cingulate cortex are reliably activated to drug cues [1]. Other meta-analyses have demonstrated that activity in these brain regions may predict relapse across multiple substances. ([2–4] Addiction Biology). One challenge for retrospective reviews and meta-analyses however, is that different research institutions often use different drug-cue reactivity paradigms, have different inclusion/exclusion criteria, and do not analyze all of their data using the same analysis pipeline. Furthermore, even when these variables are controlled, there is a lot of individual variability in the brain response to drug-cues and that there may be drug-class specific patterns of cue-reactivity [5].

While the localization of cue-reactivity in the brain is important for many reasons, it is now particularly important as multiple fields are seeking to develop brain stimulation as a treatment tool. Recent interest has developed in whether one can attenuate this cue-elicited craving through brain stimulation techniques such as transcranial magnetic stimulation (TMS) [6–8]. The location of optimal repetitive TMS (rTMS) stimulation to attenuate cue-elicited craving, however, remains elusive.

The primary aim of this investigation was to determine the spatial variability in peak cortical activity during cue-elicited craving across a large sample of individuals that performed a standardized drug cue-reactivity task tailored to their drug of dependence (cocaine, alcohol, nicotine). This was done in a cohort of non-treatment seeking individuals dependent on cocaine only, alcohol only, or nicotine only. A secondary aim was to calculate whether observed population variability could be captured by a single site of TMS simulation or if it requires within-individual functional mapping.

## Method

### Participants

For this investigation data was aggregated from four separate investigations of drug cue-reactivity performed at the Medical University of South Carolina (MUSC) from 2012–2017. In each of these studies non-treatment drug users (chronic cocaine users (*n* = 55), heavy alcohol users (*n* = 53), and current cigarette smokers (*n* = 48)) were recruited from the Charleston, SC metropolitan area using word-of-mouth advertising and digital and print media. The recruitment, consent, and initial functional MRI scanning session for all of these studies was consistent. To be eligible participants needed to be 21–60 years old and meet criteria for nicotine, alcohol, or cocaine dependence. Exclusionary criteria were related to medical history and MRI safety including known history of neurologic disease, currently meeting DSM-IV criteria for psychiatric disease (other than substance dependence), and metal implants above the waist or history of a gunshot or shrapnel in the skin. Specifically, after the initial phone contact all individuals were invited to a screening visit wherein they provided signed informed consent approved by the MUSC Institutional Review Board and completed a series of screening assessments which evaluated their medical health, psychiatric health, and drug use history. Basic demographic and drug use history of these three groups are presented in Table 1.

**Table 1:** Demographics of the substance dependent groups

	*n* = 156 Total sample	*n* = 55 Cocaine	*n* = 48 Nicotine	*n* = 53 Alcohol
*Demographics*				
Sex	101 M, 55 F	38 M, 17 F	24 M, 24 F	39 M, 14 F
Age	36.6 (±11.6)	42.7 (±9.6)	37.1 (±11.9)	29.9 (±9.7)
Ethnicity	65 AA, 91 C	48 AA, 7 C	9 AA, 39 C	8 AA, 45 C
Education	13.6 (±2.3)	12.3 (±1.8)	13.4 (±1.8)	15.1 (±2.1)
*Substance use profile*				
Cocaine used in last 30 days (%)		55 (100)	0	0
Nicotine Cigarettes used in last 30 days (%)		47 (85)	48 (100)	19 (36)
Nicotine severity (Fagerström)		3.5 (±2.4)	4.4 (±2.2)	2.1 (±2.7)
Alcohol consumed in last 30 days (%)		55 (100)	48 (100)	53 (100)
Alcohol use severity (AUDIT)		11 (±7.7)	2.4 (±2.4)	17.5 (±6.2)
Marijuana used in last 30 days (%)		23 (42)	0	9 (17)
*Mood assessment*				
Depressive symptoms (BDI)	9.5 (±11.0)	12.0 (±10.45)	2.54 (±3.2)	8.39 (±11.7)
State anxiety (STAI-S)	35.0 (±13)	36.7 (±12.4)	26.5 (±10.3)	35.1 (±14.9)
Trait anxiety (STAI-T)	37.8 (±14.7)	41.3 (±12.0)	28.5 (±7.5)	37.5 (±17.2)

Values either indicate mean (±standard deviation) or count (percent%).

*M* male, *F* female, *AA* African-American, *C* Caucasian, *MJ* marijuana, *AUDIT* alcohol use disorders identification test, *BDI* Beck's depression inventory, *STAI* Spielberger state-trait anxiety inventory.

Following the screening visit, all participants were invited to a second visit wherein they would receive an MRI scan assessing their neural response to drug cues tailored to their stated drug of choice (cocaine, alcohol, or cigarette). All participants were asked to refrain from using cocaine or alcohol on the day of the MRI scanning session and smoking cigarettes 2 h before the scanning session. Urine drug screens were used to verify abstinence from cocaine. Exhaled carbon monoxide levels were measured with a Micro-Smokelyzer (Bedfont Scientific Ltd., Kent, UK) and exhaled alcohol was measured using Breathalyzer (BACTrack).

### Drug/alcohol cue reactivity fMRI task

The drug-cue reactivity task was based on prior work [9,10,]. In the MRI environment participants viewed blocks of cocaine, alcohol, or smoking cues and neutral pictures (e.g. pencils, dishes) color matched for hue, brightness, and contrast. These task blocks were interleaved with control blocks (fixation cross, blurred images). The task was administered in the MRI scanner as a block design using E-Prime software (Psychology Software Tools, Inc.). The total task time was 12 mins and consisted of six 120-second epochs. Each epoch included alternating 24-second blocks of four task conditions: Drug, Neutral, Blur, and Rest. Respectively, these task conditions included images of cocaine- or alcohol-related stimuli customized for each group (e.g. crack pipe for cocaine users; liquor bottles for alcohol users); neutral stimuli (e.g. glass of water, cooking utensils, people eating dinner); blurred stimuli acting as visual controls by matching substance images in color and hue; and a fixation cross for alert rest periods. During each task block, 5 images were presented (4.8 s).

### Image acquisition

High-resolution T_1_-weighted anatomical images were acquired for each participant (3.0 T Siemens Trio, 3D SPGR, TR = 10 ms, TE = 3 ms, voxel dimensions 1.0 × 1.0 × 1.5 mm, 256 × 256 voxels, 124 slices). The head was positioned along the canthomeatal line. Foam padding was used to limit head motion. T2* weighted imaging data were acquired during the 12 min cue-reactivity task (TR = 2.2, TE = 35 ms, 64 × 64, 3 mm isotropic voxels).

### Neuroimaging data analysis

MRI data were preprocessed using SPM12 (Wellcome Department of Cognitive Neurology, London, UK) implemented in Matlab 7.14 (MathWorks, Inc., Natick, MA). MR Images were first converted from DICOM format to 4D NIfTI files and motion corrected (Realign: 6 parameter rigid-body realignment to first image in each timeseries using a least-squares approach). Normalization parameters, bias correction and anatomical tissue maps were determined simultaneously, using the Segment toolbox. Individual anatomical images were stripped of their skulls by masking the bias-corrected image with the combined tissue masks of gray matter, white matter, and CSF. The functional images derived from realignment were coregistered, through the mean image, to the skull-stripped anatomical image (Coregister: Estimate, using normalized mutual information). Coregistered images were then normalized (Normalize: Write) to MNI template space with the nonlinear warps derived from the Segment tool. Finally, functional images were masked (to remove the skull) and smoothed (8 mm FWHM Gaussian kernel) to facilitate subsequent analysis. Inspection of motion correction parameters revealed that all corrections were <2 mm.

Analyses were done on individual level and a group level. Overall effects were calculated using second-level, random-effects analyses of this contrast for all individuals, with cocaine, alcohol, and nicotine users each represented as a unique column in the design matrix. For each participant, first-level, fixed-effects comparisons were made to determine activation during drug/alcohol cue blocks relative to neutral blocks using the general linear model. Motion parameters (6 dimensions: *x*, *y*, *z*, yaw, pitch, roll) were included as covariates in the model. Voxel-wise correction for multiple comparisons was done via AFNI’s 3dClustSim with the autocorrelation module enabled (2017 version; https://afni.nimh.nih.gov/pub/dist/doc). Clusters with a *p*-value < 0.05 are reported (determined by Monte Carlo simulation; voxel-level threshold of *p* < 0.005 for at least 48 contiguous voxels). To investigate individual variability, the primary locus of activity in the during drug-related cues relative to neutral cues (“hot spot”) was isolated for each individual by locating the local maximum voxel (*x*, *y*, *z*, MNI coordinates) within the most significant cluster of activity (*p* < 0.05, corrected at the cluster level). The analysis was limited to cortical areas as one of the aims of this study was to identify potential frontal targets for current noninvasive brain stimulation methods such as TMS. The mask included the following bilateral regions of interest extracted from the standardized WFU_Pick atlas implemented in MATLAB (https://www.nitrc.org/projects/wfu_pickatlas): anterior cingulate cortex, middle frontal gyrus, medial frontal gyrus, inferior frontal gyrus, and superior frontal gyrus (2D dilation value: 3).

Spatial dispersion of the “hot spots”, were characterized via k-means clustering (as implemented in MATLAB and cross-checked with R). K-means clustering as implemented in Matlab used the K + + algorithm [11], an iterative, data-partitioning algorithm that assigns the total number of observations (e.g. MNI coordinates for clusters significantly activated by drug-cues) to exactly one of ‘*k*’ clusters defined by centroids, where ‘*k* ‘ is chosen before the algorithm starts. The K + + algorithm is a two-phase process which uses batch and online updates to minimize the sum of point-to-centroid distances in k clusters. Specifically, following the initial random seeding of k-centroids, a distance is calculated from each point to each centroid and that point is classified as a member of a given centroid. This is reseated, and centroid locations are reassigned if a reassignment decreases the sum of the within-cluster sum of squared point-to-centroid distance. For the present study this procedure was repeated 1000 times with random seeding. This was done for the full complement of points (*n* = 261) as well as for a restricted data set limited to one cluster per individual (*n* = 103).

In this experiment we utilized *k* values of 2–10 to evaluate the possibility that there could be up to 10 unique clusters. The optimal solution was derived via the use of silhouette plots. For each cluster the centroid vales was recorded as well as the average and maximum distance of any given point to the center of the centroid. Posthoc tests were performed to determine whether the areas of peak activity for cocaine cue-reactivity, alcohol cue-reactivity, and smoking cue-reactivity were equally distributed among the centroids (Chi-square, IBM SPSS Statistics ver.19, *p* < 0.05). The distribution of gender was also investigated given prior study from our group which suggests males may have a more uniform distribution than females.

### TMS distance methods

The data from the individual level analysis (‘hotspots’) were used to determine the scalp locations (EEG 10-10 coordinates) that were closest to the greatest number of points significantly activated by drug cues. The Euclidean distances from each hotspot to EEG 10-10 system coordinates were calculated. We evaluated the percentage of hotspots that would be reached at several distances (2–5 cm) given that the penetration characteristics of rTMS are dependent on coil size. Analyses were done for the full complement of participants as well as subgroups defined by the individual’s drug of choice.

### Code availability

Computing programs and customized scripts used in this experiment are all available for free to the community by contacting the corresponding author.

## Results

### Brain reactivity to drug/alcohol versus neutral cues—group level analysis

**Fig. 1:**
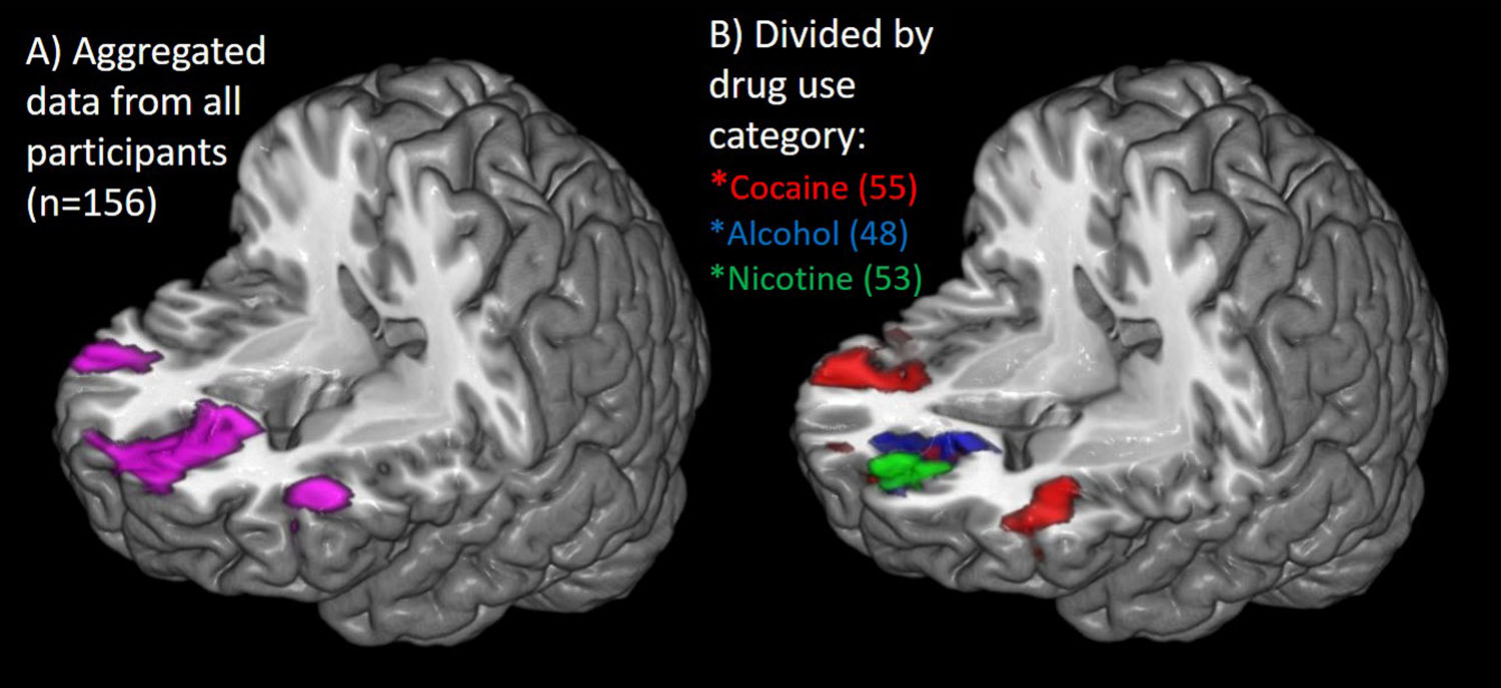
**Variability in peak location of craving**. Locations of peak activity during a cue-induced craving task were isolated for the total sample (**a**) and independently for each drug-dependent group (**b**). Considered as a group (**a**), individuals had significantly more activity in the medial prefrontal cortex, and left (BA 44) and right (BA 8) lateral prefrontal cortices during drug–related pictures relative to neutral pictures. When each group was assessed independently (**b**) the location of peak activity was concentrated in the the medial prefrontal cortex/cingulate gyrus for the alcohol users (blue) and nicotine users (green). The cocaine users had significant clusters of activity in all three of the areas activated in the total group map (BA10, left BA 44, right BA8; red)

**Fig. 2:**
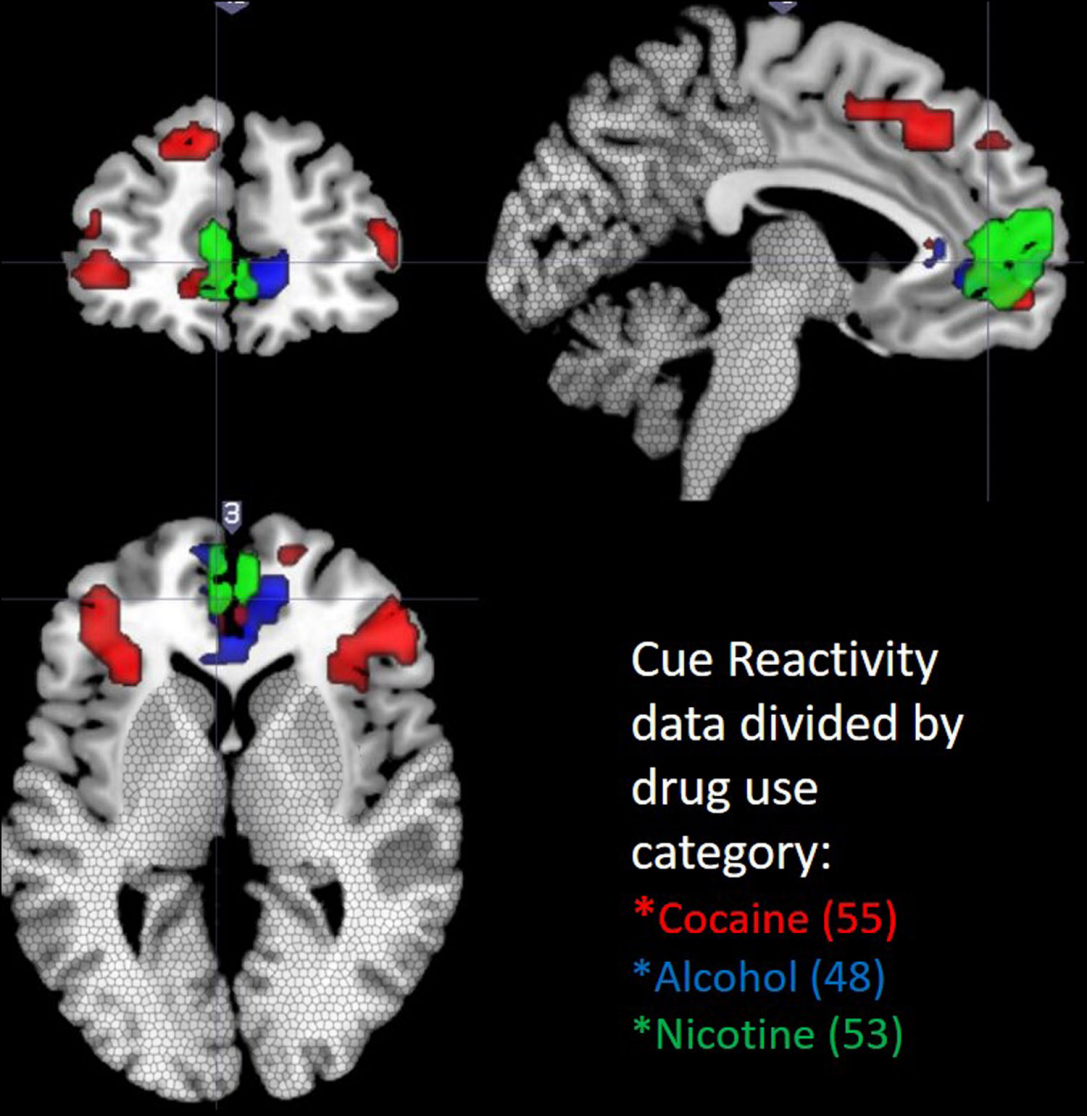
**Drug class specific topography**. In cocaine users (*n* = 55; red), cocaine cues led to significantly more BOLD signal than neutral cues in 3 clusters: (1) Left Brodmann 6 (−39,5,29; 1332, *p* < 0.000), (2) Right Brodmann 44 (45,8,29; 1057; *p* < 0.000), (3) Left Brodmann 10, ventral MPFC (−6,56, −10; 165, *p* = 0.039). In alcohol users (*n* = 53; blue) alcohol cues led to a significant increase in BOLD signal relative to neutral cues in 1 cluster: Brodmann 24/32, anterior cingulate (1,32,8; 300; *p* = 0.008) which extended through the MPFC. In cigarette smokers (*n* = 48; green), cigarette cues also led to an increase in BOLD signal in 1 cluster: (1) Brodmann 24/32, anterior cingulate (−1, 36, 10; 210; *p* = 0.048) which extended through the MPFC. The areas not included in the data analysis are shown with stippled boxes

In the population as a whole (*n* = 156), drug/alcohol cues led to a significant increase in BOLD signal relative to neutral cues in three distinct clusters, given as Brodmann Area, name (xyz coordinate; cluster size, uncorrected *p*-value): (1) Left Brodmann 10, medial prefrontal cortex (−3, 53, −4; 1253; *p* < 0.001), (2) Right Brodmann 44, inferior frontal gyrus/pars opercularis (51,11,26; 216; *p* = 0.02), 3) Left BA 8, premotor cortex (−18, 41, 41; 147; *p* = 0.05) (Fig. 1a). In alcohol users (*n* = 53) alcohol cues led to a significant increase in BOLD signal relative to neutral cues in 1 cluster: Brodmann 24/32, anterior cingulate (1,32,8; 300; *p* = 0.008) which extended through the MPFC. In cigarette smokers, cigarette cues also led to an increase in BOLD signal in 1 cluster: (1) Brodmann 24/32, anterior cingulate (−1, 36, 10; 210; *p* = 0.048) which extended through the MPFC. In cocaine users, cocaine cues led to significantly more BOLD signal than neutral cues in 3 clusters: (1) Left Brodmann 6 (−39,5,29; 1332, *p* < 0.001), (2) Right Brodmann 44 (45,8,29; 1057; *p* < 0.000), (3) Left Brodmann 10, ventral MPFC (−6,56, −10; 165, *p* = 0.039) (Fig. 1b). A more comprehensive view of the spatial topography is displayed in Fig. 2 and the whole brain data is displayed in Supplementary Figure S1 (all 156 individuals) and Figure S2 (individuals divided by substance dependence group).

### Brain reactivity to drug/alcohol versus neutral cues—individual level analysis

**Fig. 3:**
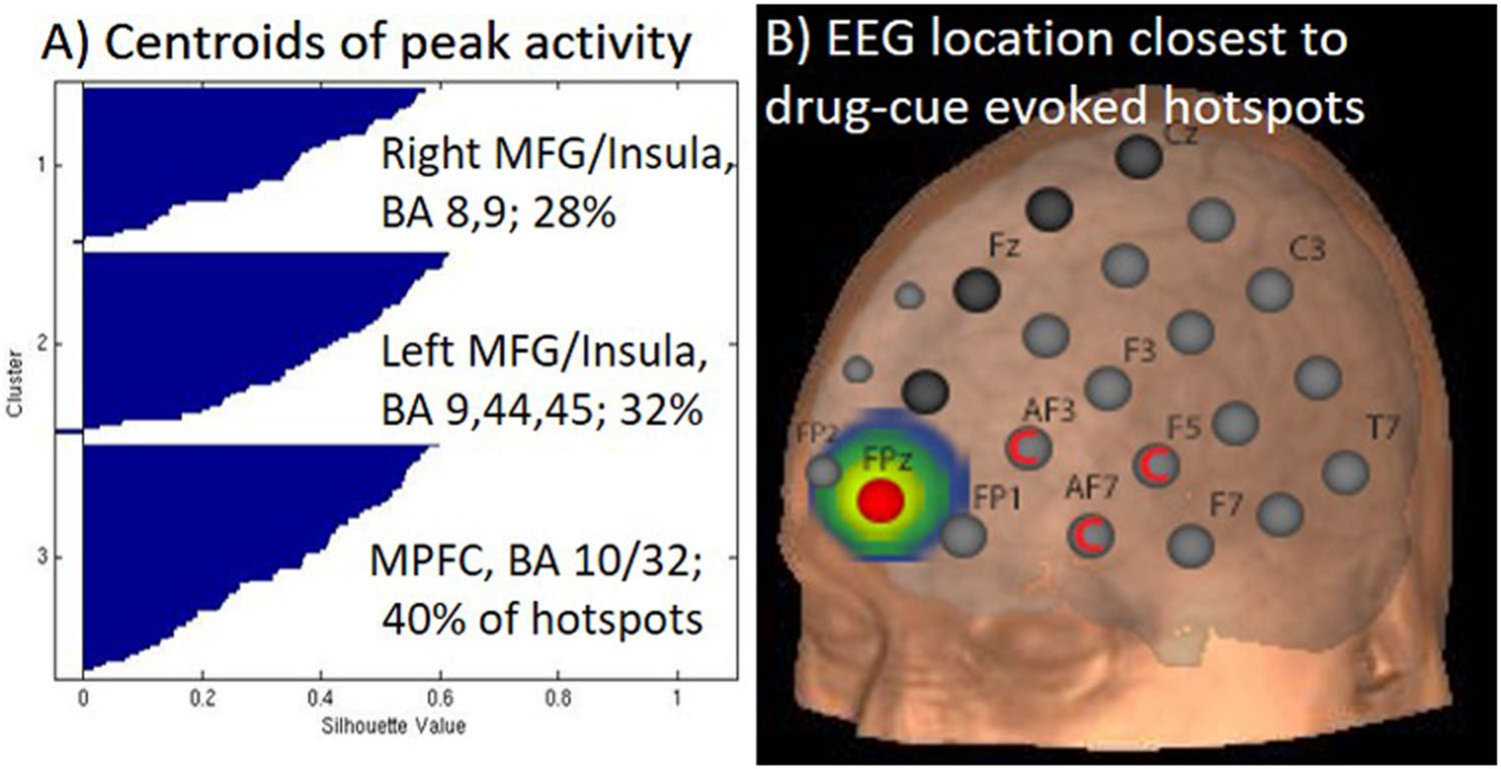
**Cluster analysis of peak craving locations**. **a** Silhouette plot of the optimal hotspot clustering solution. Spatial dispersion of the hotspots were characterized via k-means clustering (MATLAB, k + + algorithm cross-checked with R; 1000 repeats, random seeding, 1–10 clusters evaluated). K-means clustering for the full complement of hotspots revealed an optimal solution at 3 clusters (MNI Coordinates (*x*,*y*,*z*): 30, 18, 41; −40, 24, 25; 7, 50, 4). The cocaine, alcohol, and tobacco subgroups were evenly distributed through the clusters. **b** When considered as full complement, the EEG 10-10 coordinate FPz had the largest percentage of hotspots within a 2 cm (11%, red), 3 cm (19%, yellow), 4 cm (32%, green), and 5 cm (49%, blue) distance. This was also the best location for alcohol cues and smoking cues. The hotspots associated with cocaine cue-reactivity were closest to AF3, AF7, and AF5 (Red C)

Of the entire sample of 156 individuals, 103 had at least 1 cluster which was significantly elevated to the drug versus neutral cues (41 of 55 cocaine (74%), 32 of 53 alcohol (60%), 30 of 48 nicotine (63%)). K-means clustering for the full complement of points revealed an optimal solution at 3 clusters which were centered in the MPFC/ACC cortex (Cluster 1: MNI coordinates: 7, 50, 4; 40% of points, Brodmann Areas (BA): 10, 32), left lateral prefrontal cortex (Cluster 2: MNI: −40, 24, 25; 32% of the points, BA: 9,44,45, 46) and right lateral prefrontal cortex (Cluster 3: MNI: 30,18,41; 28% of the points, BA: 8,9) (Fig. 3a). There was no difference in the likelihood of classification based on drug-cue reactivity group (cocaine, alcohol, cigarettes). There was also no difference in the distribution of males and females between the clusters.

### TMS distance results

For the group as a whole, the EEG 10-10 coordinate FPz had the largest percentage of hotspots within a 2 cm (11%), 3 cm (19%), 4 cm (32%), and 5 cm (49%) distance. FPz was also the best location for alcohol cues and smoking cues (Fig. 3b, Table 2). The hotspots associated with cocaine cue-reactivity were closest to AF3, AF7, and AF5 likely driven by points in the anterior insula.

**Table 2:** The percentage of hotspots within several fixed distances from the closed cortical location as defined by the Euclidean projections of EEG 10-10 coordinate system (a system often used for targeting neuromodulation strategies)

	2 cm	3 cm	4 cm	5 cm
All hotspots	11% FPz	19% FPz	32% FPz/FP2	49% FPz
*Subgroups*:				
Cocaine cues	17% AF7/F5	27% AF7/F5	34% AF3/F5	53% AF3
Alcohol cues	17% FPz	21%FPz/FP1	36% FPz/FP2	50% FP2/AF3
Tobacco cues	10% FPz	28% FPz	45% FPz/FP2	60% FPz

## Discussion

Understanding the common or divergent patterns of drug-cue reactivity in multiple substance dependent populations is particularly important given the growing momentum for developing a neural-circuit based treatment (e.g. rTMS) for cue-evoked craving in cocaine, alcohol, and nicotine dependent individuals. Here, for the first time, we report the results of a large prospective evaluation of cue-reactivity in substance dependent individuals that all performed a standardized drug-cue reactivity paradigm with the stimuli tailored to their drug of choice. This study demonstrates that the medial prefrontal & anterior cingulate cortex are the most consistently activated clusters in cocaine cue-reactivity, alcohol cue-reactivity, and cigarette cue-reactivity – with the left and right lateral prefrontal cortices including the insula also being consistent concentrations of cue-evoked activity. When placed in the context of their proximity to putative cortical locations for brain stimulation delivery, the frontal pole is the closest location to the highest percentage of points in all populations.

### Transdiagnostic consistency of the MPFC/ACC as an area evoked by drug-cues

Data from this investigation suggest that when targeting craving circuitry with brain stimulation protocols there are at least three locations that could be targeted. The highest likelihood is the medial prefrontal cortex. When all significant hot spots were included in the analysis, 40% of these were in this MPFC/ACC cluster. This high prevalence is consistent with prior studies in the field. A meta-analysis of alcohol users, for example, evaluated 28 alcohol cue-reactivity studies. They demonstrated that among alcohol users alcohol cues consistently elicited activation in the ventral striatum, anterior cingulate and ventral medial prefrontal cortex [1]. A similar meta-analysis of smoking studies evaluated 11 smoking cue-reactivity studies. They found that smoking cues reliably evoke larger fMRI responses than neutral cues in the visual system (consistent with Hanlon et al. [12]), precuneus (consistent with Courtney et al 2014), cingulate cortex, medial prefrontal cortex, insula, and the dorsal striatum [13]. The first and only meta-analysis to evaluate common and divergent patterns of cue-reactivity across alcohol, smoking and cocaine users demonstrated that activity in the anterior cingulate cortex and striatum was the common feature across drug groups [14]. The compatibility of the results from the present study with these previous large metanalyses are encouraging, as one of the challenges of meta-analyses is that the investigators often do not have access to the raw data. Consequently, they rely upon the details reported by the individual study authors (who typically use slightly different paradigms, MRI scanning protocols, statistical processing packages, etc). In the present study we demonstrate that in a large cohort of individuals that performed a standardized drug cue reactivity paradigm on a given MRI scanner, using the same preprocessing pipeline, and a task that differed only in the drug-cue, there was consistent activity in the medial prefrontal cortex. This consistency furthers the notion that this is a transdiagnostic feature of the addiction phenotype, rather than a finding which is isolated to a specific drug using group.

### Consistent variability within each drug-cue reactivity group: left and right lateral prefrontal cortex

One of the most surprising results from this investigation was the remarkably consistent distribution that each of the drug using groups had into each of the clusters. The k-means algorithm did not take drug-use class into account for the classification. There was an unbiased distribution of the points into clusters. The optimal solution was 3 clusters. It was after those clusters were identified and the point classified that the drug use assignments and genders were assigned to the index of each point. In doing so it became clear that the data was distributed equally amongst the points for each drug class. This distribution did not change when the results were restricted to 1 point per individual.

### Implications for brain stimulation

This large individual variability may provide insight into the inconsistent outcomes of previous research using TMS for cue-induced craving. An early study demonstrated that one session of 20 Hz stimulation to the left dorsolateral PFC (LDLPFC) had no influence on craving [8]. In contrast, Amiaz and colleagues [6] demonstrated that 10 sessions of stimulation (20 Hz) to the LDLPFC led to significant, though transient, reductions in craving. A recent study selected the medial superior frontal gyrus as a target for stimulation based on functional imaging data [15]. Their group then demonstrated that 10 Hz stimulation to this area, which overlaps with Cluster 1 in our study, increased craving to smoking cues [7]. Although individual variability in the craving hot spot observed in our investigation may contribute to these mixed results, it is still unclear whether targeting the hot spots for craving directly will maximize therapeutic efficacy.

Although most neuroimaging investigations of cue-elicited craving are interested in patterns of neural activity that characterize a population as a whole, the results of this study demonstrate that there is a large variance in the location of peak brain activity during cue-elicited craving. While the craving hot spots were clustered around the mPFC in 62% of these smokers, hot spots for 38% of the population (predominantly women) were outside of this area. Although acquiring functional imaging data before brain stimulation intervention is more expensive and time-consuming, these data suggest individual imaging may be advantageous for tailoring treatment location or to filter participants before the clinical intervention.

### Limitations

In order to provide some common framework to compare this study to other studies, the anatomical data from all participants was spatially normalized to a standard anatomical template (MNI). Although there was minimal spatial distortion in this cohort, normalization compromises the spatial precision of these data. Additionally, this cohort contained a large range of cigarettes smoked per day (8–40), years of smoking (2–40) and age (20–55 years). It is possible that the individual variability of “hot spots” would be lower with a more uniform cohort.

It is also possible that these results from non-treatment seeking individuals will not broadly generalize to treatment-seeking smokers, cocaine users, and alcohol users. Cue-reactivity among treatment-seeking individuals may be different than non-treatment seeking individuals [16–18]. Additionally, it is important to note that the relationship between being treatment-seeking, receiving treatment, and stopping drug use varies between drug classes. For example, treatment-seeking alcohol users often stop drinking alcohol all together before they enter intensive outpatient treatment. Treatment seeking tobacco users, however, are typically instructed to continue smoking for several weeks after they initiate pharmacotherapy (e.g. varenicline). These treatment-related variables may also effect the temporal progression of cue-reactivity in each of these groups. When moving brain stimulation forward as a putative tool to dampen drug-cue reactivity in treatment-seeking individuals the differences in current standards of treatment will all have to be considered as important treatment variables.

### Summary

The results of this study provide critical information on the spatial distribution of craving “hot spot” in substance abusers. Moving forward it will be important to determine whether one should choose to stimulate the primary site of craving directly or to apply rTMS to neighboring regions. More explicitly, should we (1) stimulate at the site of the ‘hot spot’ to *push* the signal down in that area or should we (2) stimulate in a neighboring neural circuit to *pull* the activity away from the ‘hot spot’? From one perspective, we might expect to get maximal, sustained attenuation of cue-induced craving with an extended course of repetitive TMS directly over the hot spot. Due to the high spatial variability in the locus of peak cue-reactivity, the effectiveness of a stimulation therapy may be maximized by using functional imaging to tailor the TMS focus for each individual. Alternatively, one might choose a scalp location that will work to stimulate craving networks in 68% of individuals, and then use image guidance in non-responders.

Cue associated craving is one of the most well established, transdiagnostic markers of addiction. Many studies have assessed the spatial topography of cue-associated neural activity in specific drug classes independently. This is the first study to assess the spatial distribution of drug cue-evoked brain activity in multiple substance dependent groups who performed a standardized drug-cue reactivity paradigm at the same research center using cues tailored to their drug of choice. Considered together these data suggest that there are at least three “hot spots” consistently observed in cocaine, alcohol, and nicotine using individuals. These data may be used to inform target selection for brain stimulation treatment development which seeks to attenuate engagement of these circuits during drug cue exposure.

## Electronic supplementary material

Supplementary Data
