# Supplementary material for: Cortical substrates of cue-reactivity in multiple substance dependent populations: transdiagnostic relevance of the medial prefrontal cortex
Source: Transl Psychiatry. 2018 Sep 7;8:186. doi: 10.1038/s41398-018-0220-9 (PMC6128822; doi:10.1038/s41398-018-0220-9)

Supplementary Data

Figure S1. This is an expanded version of the data in Figure 1A which demonstrates the full data set included in the analysis (17 transverse slices shown). For each participant, first-level, fixed-effects comparisons were made to determine activation during drug/alcohol cue blocks relative to neutral blocks using the general linear model. As one of the aims of this study was to identify potential frontal targets for current noninvasive brain stimulation methods such as TMS, the analysis was limited to cortical areas. The mask included bilateral regions of interest extracted from the standardized WFU_Pick atlas implemented in MATLAB (https://www.nitrc.org/projects/wfu_pickatlas): anterior cingulate cortex, middle frontal gyrus, medial frontal gyrus, inferior frontal gyrus, superior frontal gyrus (2D dilation Value: 3). Areas not included in the mask are shown (stippled pattern). Of the entire sample of 156 individuals, 103 had at least 1 cluster which was significantly elevated to the drug versus neutral cues (41 of 55 cocaine (74%), 32 of 53 alcohol (60%), 30 of 48 nicotine (63%)). Considered as a group, individuals had significantly more activity in the medial prefrontal cortex, and left (BA 44) and right (BA 8) lateral prefrontal cortices during drug–related pictures relative to neutral pictures.


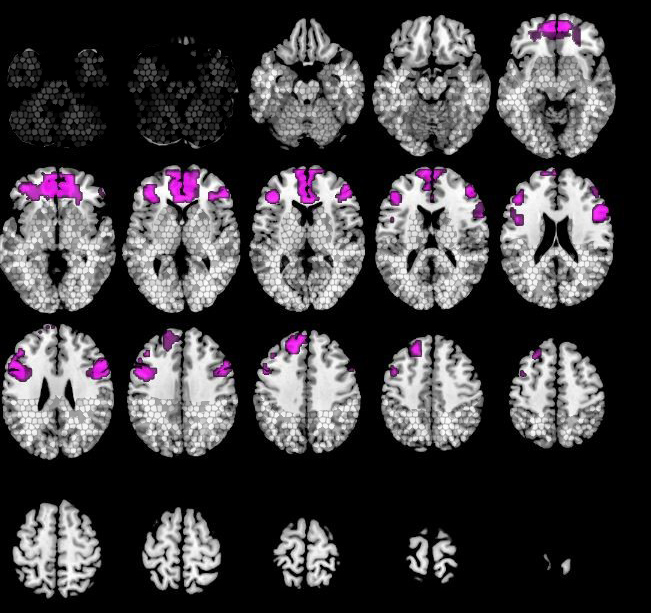


Figure S2. This is an expanded version of the data in Figure 2 which demonstrates the full data set divided by drug-using class (17 transverse slices shown). In cocaine users (red), cocaine cues led to significantly more BOLD signal than neutral cues in 3 clusters: 1) Left Brodmann 6 (p<0.001), 2) Right Brodmann 44 (p<0.0001), 3) Left Brodmann 10, ventral MPFC p=0.039). In alcohol users (blue), alcohol cues led to a significant increase in BOLD signal relative to neutral cues in 1 cluster: Brodmann 24/32, anterior cingulate (p=0.008) which extended through the MPFC. In cigarette smokers (green), cigarette cues also led to an increase in BOLD signal in 1 cluster: 1) Brodmann 24/32, anterior cingulate (-1, 36, 10; 210; p=0.048) which extended through the MPFC.


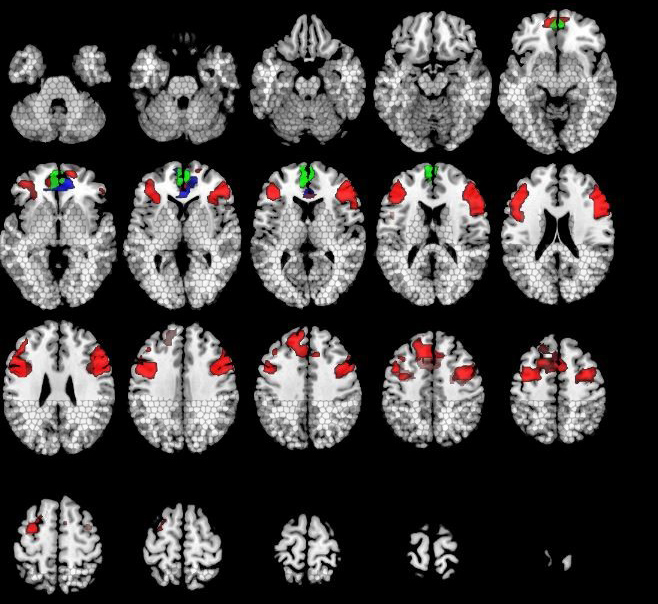

Supplement: Supplementary file 1 — Supplementary Data [file 41398_2018_220_MOESM1_ESM.docx]
